# Occurrence of β-*N*-methylamino-l-alanine (BMAA) and Isomers in Aquatic Environments and Aquatic Food Sources for Humans

**DOI:** 10.3390/toxins10020083

**Published:** 2018-02-14

**Authors:** Emilie Lance, Nathalie Arnich, Thomas Maignien, Ronel Biré

**Affiliations:** 1 UMR SEBIO, Bat 18, Campus du Moulin de la Housse, BP 1039, 51687 REIMS CEDEX 2, France; 2 ANSES—French Agency for Food, Environmental and Occupational Health & Safety, Direction de l’Evaluation des Risques, 14 rue, Pierre et Marie Curie, 94701 Maisons-Alfort, France; nathalie.arnich@anses.fr (N.A.), thomas.maignien@anses.fr (T.M.); 3 Université Paris-Est, ANSES, Laboratory for Food Safety, F94701 Maisons-Alfort, France; ronel.bire@anses.fr

**Keywords:** BMAA, seafood, freshwater foodweb, human health risk assessment, analytical methods

## Abstract

The neurotoxin β-*N*-methylamino-l-alanine (BMAA), a non-protein amino acid produced by terrestrial and aquatic cyanobacteria and by micro-algae, has been suggested to play a role as an environmental factor in the neurodegenerative disease Amyotrophic Lateral Sclerosis-Parkinsonism-Dementia complex (ALS-PDC). The ubiquitous presence of BMAA in aquatic environments and organisms along the food chain potentially makes it public health concerns. However, the BMAA-associated human health risk remains difficult to rigorously assess due to analytical challenges associated with the detection and quantification of BMAA and its natural isomers, 2,4-diamino butyric acid (DAB), β-amino-*N*-methyl-alanine (BAMA) and *N*-(2-aminoethyl) glycine (AEG). This systematic review, reporting the current knowledge on the presence of BMAA and isomers in aquatic environments and human food sources, was based on a selection and a score numbering of the scientific literature according to various qualitative and quantitative criteria concerning the chemical analytical methods used. Results from the best-graded studies show that marine bivalves are to date the matrix containing the higher amount of BMAA, far more than most fish muscles, but with an exception for shark cartilage. This review discusses the available data in terms of their use for human health risk assessment and identifies knowledge gaps requiring further investigations.

## 1. Introduction

The non-proteinogenic amino acid β-*N*-methylamino-l-alanine (BMAA) has been suggested to be a causative agent of the neurodegenerative disease Amyotrophic Lateral Sclerosis-Parkinsonism-Dementia complex (ALS-PDC) that occurred in high incidence in the South Pacific island of Guam during the 1950s [1]. BMAA, when conjugated with bicarbonate ions HCO_3_^−^ at physiological concentrations, forms carbamate adducts acting as agonists of ionotropic and metabotropic receptors of the neurotransmitter l-glutamate [2,3,4], supposedly contributing to neuronal excitotoxicity, and further motoneuron death as observed in ALS disease [5,6]. The toxic effects of BMAA on motoneurons may also be linked to an inhibition of the cysteine/glutamate antiporter, leading to a depletion of glutathione and a subsequent increased oxidative stress [7,8]. BMAA has been suggested to bind to or incorporate into proteins during their synthesis, which could induce protein misfolding and cell dysfunction [9]. However, this hypothesis has been criticized and BMAA may only be chemically associated to proteins rather than integrated into [10,11].

The consumption of flying foxes *Pteropus mariannus* has been suggested as the BMAA contamination pathway of the Chamorro population on Guam Island [12]. According to the authors, these large bats might bioconcentrate BMAA from the consumption of seeds of the cycad tree *Cycas circinalis*, which symbiont is the BMAA-producing cyanobacterial genus *Nostoc* sp. in the coralloid roots [13,14]. The first assumption of a BMAA production by the majority of cyanobacterial species, based on an analysis of 21 genera and 23 species from various terrestrial and aquatic ecosystems [15,16], has been further challenged in relation to the non-specific analytical method used [17,18,19]. Indeed, the identification of BMAA using liquid chromatography (LC) or gas chromatography (GC), associated to ultraviolet, fluorescence spectroscopy or single mass spectrometry (MS) for detection, is only based on the retention time and the signal of parent ion and may give false positive results. Particularly, BMAA can be confounded with interfering compounds eluting closely, such as its seven natural isomers (i.e., DAB: 2,4-diaminobutyric acid; BAMA: β-amino-*N*-methyl-alanine; AEG: *N*-2(aminoethyl)glycine; DABA: 2,3-diaminobutyric acid; 3,4-diaminobutyric acid; 3-amino-2-(aminomethyl)-propanoic acid; and 2,3-diamino-2-methylpropanoic acid), for which very few toxicological data and no commercial standard for the last three are available [20]. The analytical controversy that rose from 2008 enabled to determine that only the use of LC-MS/MS, with or without previous derivatization, ensures a reliable BMAA identification [18], via the follow up of specific ions generated after applying collision energy and of the ion ratio. More recent studies using specific analytical methods report the absence of BMAA in some laboratory cultures of cyanobacteria [19,21,22,23,24,25]. The BMAA production by few cyanobacterial strains has nevertheless been confirmed using specific methods [26,27,28], but at lower concentrations compared to those reported in previous studies [15,16]. To date, no screening of a high amount of cyanobacterial strains has been realized with a selective method of detection and quantification. Similarly, environmental factors or culture conditions that may influence BMAA production are still poorly understood, except for the nitrogen concentration in the medium that might modify BMAA biosynthesis [29,30], but not for all species [17]. Moreover, recent studies showed that, beside the cyanobacteria, some diatoms and dinoflagellates produce BMAA and its isomers DAB and AEG [15,16,17]. These phytoplankton species are globally distributed, and their proliferations are predicted to increase due to climate change [31], thus potentially expanding the presence of BMAA and isomers in various aquatic environments.

The presence of BMAA in phytoplankton samples has been reported in various countries such as France, Netherlands, UK, Canada, South Africa, or USA [24,32,33,34,35,36]. Some studies suggested the aerosol as a pathway of BMAA exposure of people living, or performing recreational activities, close to contaminated by BMAA-producers [37,38]. Another exposure pathway may be the consumption of aquatic organisms containing BMAA, as demonstrated for filter-feeding shellfish, crustaceans, and fish in fresh, brackish or marine waters around the world [24,39,40,41,42]. Some cases of high ALS incidence in various countries (e.g., Guam, United States, and France) have been linked to a possible long-term consumption of BMAA-contaminated food [41,43,44]. The implication of a potent BMAA biomagnification in the Guam terrestrial food web as a dietary factor causing ALS disease is still debated [45]. The presence of BMAA in primary producers and in organisms of higher trophic levels is reported, but there is no strong evidence of the BMAA trophic transfer. The degree of robustness of this potent epidemiological link through dietary intake has been recently evaluated by the French Agency for Food, Environmental and Occupational Health & Safety (ANSES) [46], and could not be validated due to the lack of available data. The hypothesis of BMAA exposition as a favoring factor of neurotoxicity and neurodegenerescence was qualified as highly probable.

To date, the level of health concern for the presence of BMAA and isomers in aquatic ecosystems or edible aquatic species remains difficult to assess in relation with uncertainties concerning the analytical methods of quantification used [18,21]. Beside a highly selective analytical method, the extraction of various BMAA fractions from samples is also required, or at least the indication of which fraction has been considered [13]. Indeed, BMAA is present in animal tissues as a soluble free amino acid (“free BMAA”), extracted with polar solvents from the sample supernatant after protein precipitation. BMAA is also associated to unknown molecules (“bound BMAA”) and extracted by a hydrolysis of: (i) the sample supernatant (“soluble bound BMAA”) in which BMAA might probably be associated to peptides and proteins of low-molecular weight staying in solution; and (ii) the pellet (“precipitated bound BMAA”) in which BMAA might probably be associated to proteins of high-molecular weight [21]. A hydrolysis of the supernatant after protein precipitation allows to quantify the “total soluble” (free + soluble bound) fraction, a hydrolysis of the pellet allows quantifying the “precipitated bound” fraction, and a hydrolysis of the whole matrix (supernatant and pellet) allows quantifying the “total BMAA” (soluble free + soluble bound + precipitated bound) content of a sample. However, very few studies considered all these fractions and more often the total and the free BMAA, and sometimes only the precipitated-bound BMAA, referred as the “protein-bound BMAA”, are reported.

The aim of this review is therefore to report the current knowledge of the presence of BMAA, and of its isomers DAB, AEG and BAMA, under various fractions when the information is available, in aquatic environments and food sources for humans, by selecting the scientific literature according to the analytical methodology used. A score system was used to select data of BMAA and isomers occurrence with intent to use it in risk assessment processes. The A from C scoring of the screened papers was based on the number of qualitative and quantitative criteria (e.g., retention time, specific transitions of BMAA and its isomers and ion ratios, the indication of the limit of detection (LOD) and limit of quantification (LOQ), the calculation of BMAA or isomers recovery in matrix, and the evaluation of matrix effects) that the analytical method fulfilled. This screening was combined with a review of current analytical approaches and their efficiency to obtain accurate BMAA quantification [46]. Only BMAA and isomers concentrations in aquatic environments and in food sources for humans originating from A and B graded studies are reported in this systematic review and data are discussed in relation to the potential risk for humans and necessary further research.

## 2. Results

**Figure 1:**
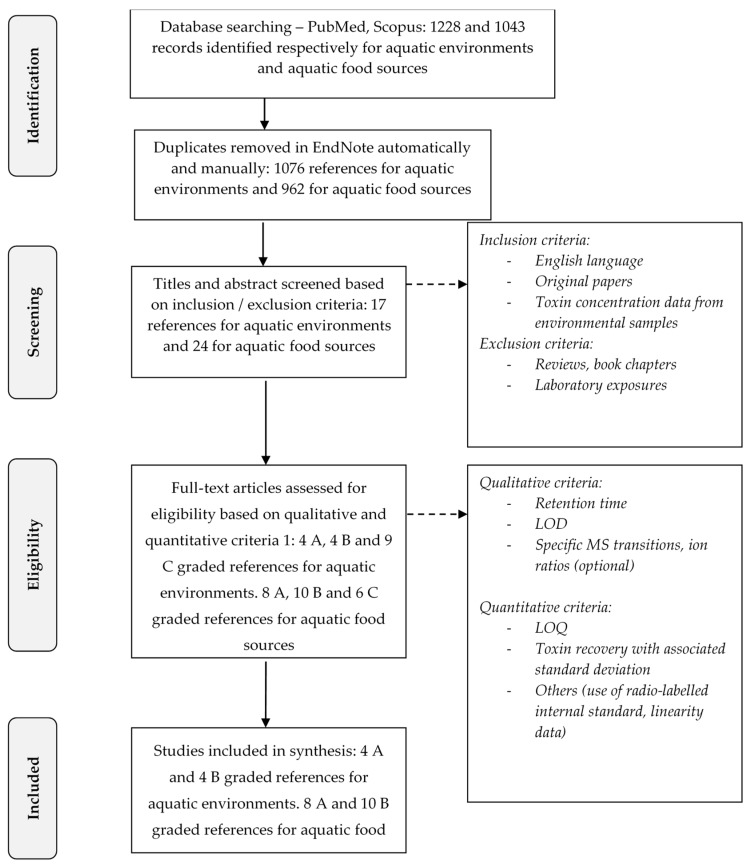
Schematic diagram presenting the systematic review process ^1^. Adapted from [47]. ^1^ Articles were graded B when quantitative criteria were not met (e.g., no LOQ or no toxin recovery data). Articles were graded C because the initial qualitative criteria were not met; the lack of specific MS transitions was one of the major rejection criteria.

The selection process of papers according to analytical methodologies used to quantify BMAA and isomers levels in aquatic ecosystems and organisms is presented in the Figure 1 and in Section 5.2. The scoring of screened studies retained in this review (A and B graded) is indicated in Table 1, Table 2 and Table 3.

### 2.1. Presence of BMAA and Isomers in Waters and Phytoplankton Samples

This review reports the BMAA and isomer concentration data obtained from A and B graded studies and concerning environmental water samples (i.e., lakes, lagoon and reservoirs), indicated in Table 1.

#### 2.1.1. Lakes and Reservoirs

In the Netherlands, free BMAA was found in 43% of samples collected in 21 urban water bodies, at concentrations reaching 42 µg g^−1^ of dry weight (DW) [34]. Free DAB was detected in 10% of the samples but reported concentrations (maximum of 4 μg g^−1^ DW) were below the LOD indicated as 4 μg g^−1^ DW by the authors (Table 1). In Sweden, one study [48] reported traces of BMAA (up to 6 ng g^−1^ DW) in 75% of samples collected in the Finjasjön Lake. In Canada, 33% of the cyanobacterial bloom samples collected in 12 lakes from the Province of Quebec between 2009 and 2013 contained BMAA at a maximum concentration of 0.3 μg L^−1^ [36]. While the study did not clearly report which BMAA fraction was analyzed, the absence of acidic extraction and the matrix water analyzed are indication that only free BMAA was considered. Traces (<0.1 μg L^−1^) of AEG and DAB were detected in 42 and 50% of the samples, respectively (Table 1). In China, neither free nor protein-bound BMAA was found in samples collected in three lakes in which the cyanobacteria *Microcystis spp*. and *Dolichospermum flos-aquae* regularly dominate phytoplankton communities. Some traces of free DAB (from 0.4 to 3.8 ng g^−1^ DW) were however detected in 100% of the samples [49].

#### 2.1.2. Lagoon, Brackish and Marine Environments

In France, 100% of periphyton samples from the Thau Lagoon, a shallow costal lagoon off the Mediterranean Sea frequently subject to phytoplanktonic blooms, contained BMAA and DAB at maximum concentrations of 4.3 and 4.8 μg g^−1^ DW, respectively [24]. Some traces of AEG were also reported in 66% of the samples (Table 1). These authors also reported the presence of both compounds in the microalgae- and zooplankton-dominated fractions of the seston originating from the Thau Lagoon, but at lower concentrations (mean concentrations < 1 μg g^−1^ DW). One study reports the presence of BMAA in 100% of the samples collected on Askö Island in the Baltic Sea, at concentrations between 2.3 and 15 ng g^−1^ DW, in association with the presence of the cyanobacterial genus *Nodularia* and *Aphanizomenon* [40].

### 2.2. Contamination of Aquatic Food Sources for Humans

Among the 20 reports of BMAA and its isomers in human food sources we selected, eight indicated LOD and LOQ, method performance criteria, quantified data and chromatograms and were then A graded; 12 did not report LOD and LOQ but satisfied qualitative criteria and were then rated “B” category. Table 2 and Table 3 report BMAA and isomer quantification data from the A and B graded studies, respectively.

#### 2.2.1. Contamination of Freshwater Organisms

● Freshwater crustaceans

A large screening of BMAA contents in seafood from Swedish markets, performed in 2013 and 2014 and graded A, reported the absence of BMAA (i.e., concentration under the LOD and LOQ of 0.01 µg BMAA g^−1^ FW) in flesh of freshwater crayfishs originating from Swedish and Turkish lakes [50].

● Freshwater fish

No A graded study reports BMAA in freshwater fish and only data from B graded studies are reported here. A single carp was caught in the Mascoma Lake (New Hampshire), a lake associated with a high ALS incidence in the population living nearby [51]. The concentrations of BMAA and DAB (total fractions) in the carp brain was 0.086 and 0.002 mg kg^−1^ FW, respectively. BMAA was also detected in the carp liver (0.26 mg kg^−1^ FW) and muscles (0.25 mg kg^−1^ FW), but not DAB (Table 3). The BMAA contents patterns in the brain, muscle, liver and kidney of several fish species from different trophic levels (e.g., plankti-benthivorous and piscivorous fish) were assessed in the Swedish Finjasjön Lake suffering from major blooms of cyanobacteria [48]. Authors reported a BMAA concentration varying from 0.00002 to 0.0016 mg kg^−1^ FW in the muscle of 16% of the fish (bream, perch, pike, pike-perch, roach, ruffe and tench, with higher percentage for bream feeding on benthic preys) (Table 3).

#### 2.2.2. Contamination of Marine Organisms

● Marine Bivalves and Crustaceans

(1) Europe

In the South of France, several villages located along the Thau Lagoon show an increased ALS incidence, coinciding with the presence of BMAA in seafood locally produced [41]. A study graded A [23] reported the presence free and total soluble (free + soluble bound) BMAA, DAB and AEG in 100% of mussel (*Mytilus galloprovincialis*) and oyster (*Crassostrea gigas*) samples from the Thau Lagoon at concentrations varying from 0.1 to 2.45 mg kg^−1^ FW (Table 2). Free BMAA was sporadically detected. In mussels, free BMAA and AEG were detected at lower concentrations (<0.34 and <0.08 mg kg^−1^ FW respectively) and less frequently (in 4 and 3 out of 11 samples, respectively) than total soluble BMAA and AEG (0.64–2.45 and 0.1–0.2 mg kg^−1^ FW, respectively) detected in all samples. The same applied to oysters: free BMAA at <0.08 mg kg^−1^ FW in one out of eight samples and AEG not detected, against concentrations of 0.5–1.8 and 0.1–0.3 mg kg^−1^ FW of total soluble BMAA and AEG reported in all samples respectively. These concentrations of total soluble BMAA are in accordance with those of total BMAA (i.e., 0.66 mg kg^−1^ FW) reported in another A graded study for oysters originating from the French Atlantic coast [50]. The total soluble BMAA and AEG contents in mussels from the Thau Lagoon showed a temporal kinetic with a gradual increase from June to September 2009, corresponding to a period of increased filtering activity of bivalves and phytoplankton proliferations [23]. DAB was reported in all mussel and oyster samples without temporal variation over the season, both under free (respectively, 0.08–1.2 and 0.03–0.6 mg kg^−1^ FW) and total soluble form (respectively, 0.6–1.6 and 0.6–1.5 mg kg^−1^ FW). The authors again investigated Thau Lagoon between July 2013 and August 2014 with monthly sampling of plankton, mussels and the biofilm on their shell, and reported the presence of BMAA, DAB and AEG in all samples [24]. In mussels, free BMAA occurred less often (16/34 samples) and at lower concentrations (max 0.2 mg kg^−1^ FW) than total soluble BMAA (max 1.65 mg kg^−1^ FW), which varied on seasonal basis with an increase in summer and autumn 2013 and 2014. The total soluble AEG was found at a maximum concentration of 0.2 mg kg^−1^ FW in 31 out of 34 samples of bivalves, but much more rarely under the free form: in 5 out of 34 samples and at maximal concentration of 0.05 mg kg^−1^ FW. However, DAB was found in all the samples and at constant concentrations in various organs during the year of investigations (average of 1.2 mg kg^−1^ FW), both in free (max 1.05 mg kg^−1^ FW) and total soluble (max 1.8 mg kg^−1^ FW) forms. The same authors further assessed the occurrence of BMAA and isomers in 97 mollusks (mussels and oysters) collected from nine representative shellfish production areas located on the three French coasts (Channel, Atlantic and Mediterranean sites) [42]. Total soluble BMAA and DAB were systematically detected in the digestive glands of bivalves in concentrations ranging from 0.03 to 1.13 mg kg^−1^ FW and 0.20 to 4.84 mg kg^−1^ FW respectively. The concentrations of total soluble BMAA and DAB were similar in mussels and oysters from the different sites during the investigation.

Marine mussels originating from the Western Coast of Sweden showed total BMAA contents varying from 0.27 to 1.6 mg kg^−1^ FW [53], being in accordance with the levels (0.64–2.45 mg kg^−1^ FW) reported in French mussels. Another A graded study [20] reports the presence of free BMAA in mussels and oysters sampled from the Western Coast of Sweden but without quantification results, and was therefore not included in the Table 2. In 2013 and 2014, the same authors investigated fish, bivalves and crustaceans sold in supermarkets and open markets of Stockholm, and reported concentrations of total BMAA of: (i) 0.08–0.9 mg kg^−1^ FW in mussels and oysters from the Swedish west coast; (ii) 0.32 mg kg^−1^ FW in oysters from Greece; and (iii) 0.11–0.46 mg kg^−1^ FW in shrimps from Sweden and Northern Atlantic [50]. Concerning the seafood originating from the Baltic Sea, low levels (from 0.02 to 0.03 mg kg^−1^ FW in blue mussels and from <LOD to 0.02 mg kg^−1^ FW in oysters) [40] were detected in marine bivalves (Table 3). A study identified total BMAA in oysters (*O. edulis*) from a Swedish market but results expressed as µg BMAA L^−1^ cannot be compared with other reports and were not included in this review [28]. Similarly, the presence of total BMAA in non-visceral and in visceral tissues of blue mussels *M. edulis* was reported without any quantification and any specification of their origin [61]. In Portugal, concentrations up to 0.08 mg kg^−1^ FW of precipitated bound BMAA were reported in cockles (*Cerastoderma edule*) from two ria of the Atlantic coast. The BMAA quantification values were correlated or not with the cell density of the BMAA-producing dinoflagellate *Gymnodinium catenatum* depending on the investigated site [56]. These levels of precipitated bound BMAA in shellfish tissues are lower than those of total or total soluble BMAA reported above (i.e., from 0.08 to 8 mg kg^−1^ FW, Table 2). The two isomers AEG and DAB were also detected in cockle tissues but not quantified.

(2) USA and Canada

The concentrations of total BMAA in oysters collected in the southeast of the United States varied from 1.5 to 8 mg kg^−1^ FW in Louisiana and from 1.2 to 1.7 mg kg^−1^ FW in Mississippi. The same authors reported total BMAA concentration varying from 1.08 to 3.02 mg kg^−1^ FW in blue crab muscles from Florida (study graded A, Table 2) [52]. Similar (up to 6.94 mg kg^−1^ FW) and higher (34.6 mg kg^−1^ FW) total BMAA concentrations were quantified, respectively, in boiled lobster originating from Florida Bay [43] and in blue crab meat (sum of total soluble and precipitated bound) originating from the Chesapeake Bay [44] (studies graded B, Table 3). According to these studies, DAB was also quantified in blue crab (from 11.6 to 15.8 mg kg^−1^ FW total soluble and from ND to 2.91 mg kg^−1^ FW precipitated bound) and lobster (from 0.09 to 7.99 mg kg^−1^ FW total) meat (Table 3). In Canada, the presence of DAB, AEG and BAMA was detected in tissues of mussels originating from markets, but only total BMAA was quantified at concentrations ranging from 0.19 to 0.24 mg kg^−1^ FW [57], comparable to those reported in A graded studies.

(3) Diverse or unknown origins

In China, 68 samples from 29 species originating from aquaculture and bought at local markets of several towns were analyzed in a study graded B [58]. The presence of free BMAA in 5 of the 68 edible mollusks from three marine species was reported: one mussel (*Mytilus coruscus* 0.45 mg kg^−1^ FW) sample out of nine, one razor clam (*Solen strictus* 0.66 mg kg^−1^ FW) out of one, and three gastropods (*Neverita didyma* up to 3.97 mg kg^−1^ FW) out of five analyzed. Eight other mussel (*M. galloprovincialis*) and 10 oyster (*Crassostrea sp*) samples collected on Chinese coasts did not contain free BMAA (Table 3). Free DAB was detected in 53 out of 68 samples from 23 marine species, oysters and mussels, at concentrations varying from 0.05 to 2.65 mg kg^−1^ FW. Free DAB concentrations were similar between species and prospected areas. Authors reported the absence of protein-bound BMAA and DAB, as well as the absence of AEG isomer regardless of the analyzed fraction. A A graded study showed that neither free nor total BMAA was detected in shrimp and crayfish bought in Swedish markets and originating from diverse continents [54], although few crustacean samples have been analyzed (*n* < 3). However, mussels (from South America and Australia), scallops (from North America) and crabs (from Europe) contained significant levels of total BMAA, i.e., from 0.28 to 7.08 mg kg^−1^ FW. Cooked and canned mussels imported from South America contained the highest total BMAA concentration (7.08 mg kg^−1^ FW), up to ten times higher than concentrations found in mussels from Scandinavia bought fresh and further cooked and frozen (Table 2). In this study, free BMAA represented a negligible part (i.e., 6.5%) of total BMAA quantified in seafood [54].

● Marine fish

(1) Europe

Low total BMAA concentrations (from 0.002 to 0.014 mg kg^−1^ FW) were detected in muscles of several fish species (smelt *Osmerus eperlanus*, turbot *Scophthalmus maximus*, herring *Clupea harengus*, pike-perch *Sander lucioperca*, fourhorn sculpin *Triglopsis quadricornis*, and whitefish *Coregonus laveratus*) from the Baltic Sea [40]. In Sweden, total BMAA was detected in whitefish (*Coregonus lavaretus*) from market but results expressed in µg L^−1^ and not in µg kg^−1^ could not be used [28] (Table 3).

(2) Diverse or unknown origins

No total BMAA was found in muscles of various fish (salmon *Salmo salar*, sea bass *Dicentrarchus labrax*, bream *Sparus aurata*, whitefish *Coregonus sp*, pike perch *Sander lucioperca*, and sea trout *Salmo truttae*) from Sweden or imported from Norway, Italy and Greece [54], but only a single sample of each fish was analyzed in this study (Table 2). The absence of total BMAA was also reported for salmon (*Salmo salar*), cod (*Gadus morhua*) and perch (*Perca fluviatilis*) from trade [50]. However, some total BMAA was quantified in plaices *Pleuronectes platessa* (in all three samples) from Sweden and in herrings *Clupea harengus* (one out of three samples) from Baltic Sea, but at low concentrations, close to the LOD and LOQ (from <LOD to 0.02 mg kg^−1^ FW) [50]. Among marine taxa, the BMAA content appears to be much higher in sharks to date. Total BMAA concentrations between 19.2 and 33.15 mg kg^−1^ FW were reported in fins and muscle of four shark species [59]. From this study, we only considered data of quantification performed by ultra-pressure liquid chromatography coupled with tandem mass spectrometry (UPLC-MS/MS) and not the one acquired by liquid chromatography with fluorescence detection (HPLC-FLD), the latter technique being less specific. In agreement with their earlier study on shark fins [60], Mondo et al. [55] further reported the presence of total BMAA in 15 out of 16 analyzed dietary supplements containing shark cartilage, from various species and from various origins (not reported). The total BMAA contents varied between 74.8 and 352.2 mg kg^−1^ DW (mean of 169 mg kg^−1^ DW). Total DAB (from 69.2 to 1483.4 mg kg^−1^ DW, mean of 318 mg kg^−1^ DW) and AEG (from 1298.4 to 1765.1 mg kg^−1^ DW, mean of 1627 mg kg^−1^ DW) were reported in all the 16 samples (Table 3). These data were not expressed in mg kg^−1^ FW due to the lyophilized form of the product consumed by humans.

## 3. Discussion

This systematic review only reports data on the occurrence of BMAA, DAB, and AEG in aquatic environments (water or phytoplankton samples) and in aquatic animal species of the food web (sampled in situ), extracted from original papers (no review or book chapter) and written in English. A drastic selection among studies was performed according to strict qualitative and quantitative criteria concerning the chemical analytical method used. The studies using LC-MS/MS and satisfying both the qualitative (e.g., retention time, ion ratio, specific transitions, and LOD) and quantitative (e.g., LOQ, recovery of the method, and use of an internal standard) criteria were graded A, those satisfying only the qualitative criteria were graded B, and those that do not fulfil any of the above criteria or do not provide sufficient information to estimate the validity of the method were graded C. This classification does not question, in any means, the quality of the experimental protocol or the sampling approach described in the papers. Only quantitative data from the A graded studies are considered as truly reliable. The detection of BMAA reported in B graded studies is also reliable but doubt subsists concerning the validity of the quantification. Considering this uncertainty and in the light of the scarcity of available data (particularly for fish), we present both A and B graded studies by discriminating them, and discuss using a maximum of A graded studies when possible.

The presence of BMAA in aquatic ecosystems supposedly originates from the proliferations of phytoplanktonic or phytobenthic species. As cyanobacteria were first considered as the main BMAA producers in fresh waters [16], most of reported studies focused on sites subjected to cyanobacterial proliferations. A few cyanobacterial species (e.g., *Leptolyngbya* sp., *Nostoc* sp.) have been shown to produce BMAA in laboratory conditions [26,27,28]. Comparisons of BMAA concentrations reported in aquatic ecosystems is difficult due to the limited amount of data and their expression per either volume or phytoplanktonic biomass. Only one BMAA concentration value expressed in µg L^−1^ (i.e., 0.3 µg L^−1^ in Canada, [36]) is available among A and B graded studies. When data are reported per unit of phytoplankton biomass, BMAA concentrations vary from 2.3 ng g^−1^ in Sweden [40] to 42 µg g^−1^ in the Netherlands [34]. For comparison, the C graded studies report BMAA concentrations varying from 2 µg L^−1^ in Canada [62] to 39.6 µg L^−1^ in USA [32], or from 0.25 µg g^−1^ of phytoplankton in South Africa [29] to 276 µg g^−1^ of phytoplankton in UK [35]. Such variability may be attributed to differences in: (i) methodologies used to quantify the metabolites; (ii) the density of BMAA producers in situ; and (iii) the varying capacities to produce BMAA depending on genus, species and even strains of producers. Only one study [24] reports qualitative (i.e., species characterization) and quantitative (i.e., cell densities or biomass) data relative to the phytoplanktonic composition of the analyzed samples, although it should be done systematically. In the Thau Lagoon, France, the authors [24] reported the presence of BMAA in seston (phytoplankton and zooplankton) samples mainly composed of diatoms (mostly *Chaetoceros* sp. producing BMAA in laboratory conditions). Indeed, beside the cyanobacteria, various diatoms (e.g., *Phaeodactylum tricornutum*, *Chaetoceros* sp., *Chaetoceros calcitrans* and *Thalassiosira pseudonana*) and dinoflagellates (e.g., *Heterocapsa triquetra*, *Gymnodinium catenatum*) produce BMAA and its isomers DAB and AEG [24,30,50,56], increasing the concern for brackish and marine environments. However, only one study investigating the marine waters was available at the date of this review [40], restricting the possibility to characterize the contamination of this medium. Diatoms are known to sustain the growth of bivalves and may be a trophic pathway for their contamination by BMAA and isomers, as already suggested [24,50,56].

The review of A graded studies show that concentrations of total BMAA in seafood and fish muscle vary from 0.02 to 8 mg kg^−1^ FW depending on the taxa, and that the marine bivalves are to date the most contaminated food source for human. All five A graded studies considering marine mussels and oysters report a positive detection of BMAA with concentrations varying from 0.08 to 8 mg kg^−1^ FW. Bivalves are suspension-feeding animals with high filtering activity, for which the BMAA bioaccumulation following the ingestion of producing microalgae has not been clearly demonstrated [63]. To date, such trophic pathway remains difficult to be laboratory investigated due to the absence of a known microalgae or cyanobacterial strain continuously producing BMAA in culture conditions. However, the neurotoxin has been found in their tissues at substantial concentrations following laboratory exposure to dissolved BMAA [64,65]. The crustaceans, considered in three A graded studies, present slightly lower BMAA concentrations in their tissues than bivalves (i.e., from LOD in crayfish to 3.02 mg kg^−1^ FW in crabs). Fish muscles appear to be the less contaminated matrix with a maximum of 0.02 mg BMAA kg^−1^ FW in two species among 12 analyzed in two A graded studies. Concerning B graded studies, low BMAA content (maximum of 0.25 mg kg^−1^ FW) are reported in the muscle of freshwater fish [48,51]. The same trend is observed for marine fish in which either no BMAA or a maximum of 0.02 mg kg^−1^ FW of total BMAA is reported [50,54]. Although data mainly originate from a B graded study, it is noteworthy to mention the high BMAA contents (up to 33.15 mg kg^−1^ FW) reported in shark fins [59]. Moreover, a study graded A reported BMAA concentrations from 74.8 to 352.2 mg kg^−1^ DW in 15 out of 16 analyzed dietary supplements containing shark cartilage [55]. The low amount of available A graded studies on fish (zero and three respectively for freshwater and marine fish) makes it difficult to analyze trends for the contamination of their edible parts, that would deserve further investigations with highly reliable methods.

To assess health risk associated with BMAA-containing food, it is of great importance that data includes all the BMAA fractions (free, soluble or precipitated-bound, or total). Most of the studies we graded A and B report the total BMAA content in edible organisms, few of them [23,24,43,44,54,58] report both the total and free BMAA contents, or both the total soluble and free BMAA contents [23,24,43,44,54,58]. In these last studies, the total soluble BMAA content far exceeds that of free BMAA (e.g., maximum of 2.45 mg total BMAA kg^−1^ FW against 0.2 mg free BMAA kg^−1^ FW in mussels) [23,24]. Moreover, the total soluble BMAA was detected in all the samples of mussels and oysters, whereas the free fraction was only detected in four mussels and one oyster among 19 samples [23]. The soluble-bound BMAA, remaining in the supernatant solution after protein precipitation may account for a major part of the total BMAA [21]. Therefore, studies [23,24] reporting the total soluble and free BMAA contents in organisms (without addressing the precipitated bound fraction) might not have overly underestimated the real BMAA content. The soluble-bound fraction, encompassed in the total BMAA measurement via sample hydrolysis, has been omitted in studies that separately measured free and precipitated bound BMAA (e.g., in reference [60]). In this case, an underestimation of the BMAA content is highly probable. The soluble-bound fraction was suggested for flesh samples of two lobsters in a study in which the supernatant was hydrolysed leading to a higher BMAA content (up to 27 µg g DW^−1^, total soluble BMAA) than in the TCA extracted supernatant of the corresponding individual (up to 0.4 µg g DW^−1^, free BMAA) [43]. However, the comparison is difficult because the TCA extraction was performed on the supernatant of non-boiled flesh whereas the hydrolysis was carried out on the supernatant of boiled one. One of the purposes of the authors was to evaluate the impact of the boiling on the BMAA flesh content. Similarly, the comparison between boiling or not for the two lobsters cannot exactly be assessed as the authors analyzed the total BMAA content (pellet and supernatant hydrolysed) for boiled parts, and only total BMAA in the pellet and soluble free BMAA in the supernatant, without the soluble-bound fraction, in the fresh parts. The inclusion of the soluble bound fraction, by measuring the total soluble fraction or by directly measuring the total BMAA content in the entire sample, is therefore mandatory for a BMAA quantification in biological matrix, as earlier suggested [21]. To date, the precursors giving BMAA after hydrolysis of protein or polypeptide fractions are still unknown [21], as well as the exact nature of the interaction between BMAA and proteins or peptides. The mechanism of BMAA association to proteins, through non-specific or non-covalent bonding, or its covalent misincorporation during the protein synthesis causing protein misfolding, aggregation and/or loss of function, have been both suggested but not clearly proved or disproved [9,66] and remains debated [11]. The results of an exposure of various freshwater mussels to dissolved labeled ^5+^BMAA suggested a metabolization via a reversible covalent modification of BMAA, variable in different species, but did not provide any evidence of BMAA association with proteins [64]. The BMAA content was evaluated in various fractions of mussel tissues with conclusion that BMAA is neither free nor bound to proteins, but rather bound to amino acids forming a low molecular weight compound [67]. The authors also mentioned the possibility of a chelation with metals or carbamate formation that can lead to different forms of bound BMAA in biological matrix. The amount of amino acids (Arg, Asp, Glu, Leu, Lys, Ser, Thr, Ile and Met) measured in muscle of a marine snail was shown to decrease concomitantly with the increase of the free BMAA content, suggesting that BMAA is not incorporated into proteins but may affect their synthesis [58]. Moreover, it was recently demonstrated that the slight toxicity of BMAA on human cell lines was not the result of its misincorporation into proteins, as no BMAA was detected in purified protein extracts [11].

Beyond the necessity to evaluate the proportions and origin of various BMAA fractions, further work is required to understand its organotropism at the individual level. Although the cyanotoxin microcystins mainly accumulate under a protein bound form in the digestive glands of invertebrates or liver of vertebrates after ingestion of primary producers [68,69], the organotropism of various BMAA fractions in invertebrates or vertebrates has not been elucidated so far. The few studies investigating BMAA content in several body parts of marine mussels, for which the exact contamination pathway is still unknown, reported a BMAA presence both in the visceral and non-visceral tissues [24,61]. A study revealed that concentrations of free and total BMAA in the digestive gland were lower than in the rest of the body (respectively 69.3 ± 17% and 59.5 ± 6%), whereas the opposite was observed for free and total AEG [24]. Identically, the BMAA distribution pattern in aquatic ecosystems has been suspected of being a bioamplification in the Florida Bay [39] but was not mentioned as such in the Baltic Sea [40], and therefore requires further in situ investigation.

Very few studies report concentrations of BMAA isomers in edible organisms. DAB (mean of 318 mg kg^−1^ DW) and AEG (mean of 1627 mg kg^−1^ DW) were found with BMAA in 16 samples of dietary supplements containing shark cartilage [55]. The studies from Reveillon et al. [23,24,30] report the presence of DAB and AEG in marine mussels and oysters as well as their production by diatoms. The DAB concentrations in bivalve tissues are of the same order of magnitude than those of BMAA, from 0.6 to 2.4 mg kg^−1^ FW. The DAB (also named DABA) is a component of some bacteria wall (e.g., genus *Corynebacterium*) [70] or actinomycetes (e.g., *Agrococcus jenensis*) [71]. The presence of DAB has been also specifically reported in angiosperm species such as *Lathyrus latifolius* or *Brassica oleracea* [19,28] as well as in aquatic [32] or terrestrial [72] plants. DAB was reported for the first time in 2008 in the cyanobacteria *Calothrix* sp. PCC7103 [25] and further detected in ten cyanobacterial genus (e.g., *Synechococcus* sp., *Leptolyngbya* sp., *Synechocystis* sp., *Nostoc* sp., *Microcystis*) in which no BMAA was found [23,24]. Similar observations were reported in 100% of the samples collected in three Chinese lakes [49]. Interestingly, in China, free BMAA was detected in five out of 68 edible species, whereas free DAB was detected in 53 of them, at similar concentrations between species and prospected areas [58]. Identically, the total BMAA and AEG contents in marine bivalves showed seasonal variations with an increase in summer and autumn (years 2009, 2013 and 2014) that was concomitant to the increased filtration activity and phytoplanktonic densities, suggesting an environmental and seasonal origin of those amino acids and a contamination by a trophic pathway. Conversely, the free and total DAB contents in bivalves were constant over the year in all samples [23,24], suggesting a more widespread origin such as an endocytosis from the medium or from producing bacteria living in/on the organisms, or a constitutive molecule of the tissues, that remain to be investigated.

## 4. Conclusions

This review highlights that the continuous presence of total BMAA in marine mollusks, with the free forms being more sporadic, suggests a potential risk for consumers. Moreover, a study [42] confirmed the systematic widespread occurrence of BMAA in shellfish from all French coasts, suggesting the need for epidemiological studies to evaluate the potent link between their local high consumption and some significant ALS clusters, such as observed in the French Thau Lagoon [41]. An accurate report of BMAA and isomer contents in edible species is crucial to evaluate a human exposure and potent associated risks. Another exhaustive literature review of human dietary exposure to BMAA [73] was recently published, but is neither systematic (e.g., some references we graded B have not been taken into account [21,40,52,54,57,58,60,62]), nor selective according to the accuracy of the identification or quantification of BMAA (e.g., the reported data on BMAA content in fish mainly derived from studies we graded C [25,32,34]). Therefore, the present systematic review offers a supplemental step based on the chemical analytical method accuracy, and is appropriate in view of a health risk assessment procedure. Nevertheless, it is not completely satisfactory, as available data remain largely incomplete and many questions remain unanswered. The BMAA contents in organisms may vary in relation with: (i) inter-specific variations; (ii) their trophic status regarding the BMAA producers; and (iii) varying environmental contamination levels depending on geographical and seasonal parameters. We mainly discussed results from A graded studies using very selective analytical methods, but most organisms including benthic feeders and top predators remain to be investigated. For instance, very few of the most important commercial fish species have been investigated and the risk associated with their consumption cannot be assessed. Moreover, several studies suggest the necessity to evaluate the total fractions of BMAA and its isomers, as the protein-bound fraction (and mostly the soluble bound) commonly exceeds the free one in biological matrix. Extensive research is required to specify the exogenous origin of BMAA, its mechanism of binding to/incorporation in proteins, its organotropism and potent bioaccumulation patterns in the food webs. Moreover, more laboratory studies are mandatory to identify BMAA and isomer producers, as well as biosynthesis pathways. Thorough environmental studies of a diversity of samplings showing contrasted phytoplanktonic populations in freshwater, brackish and marine ecosystems may also give an estimate of the BMAA-producer diversity. The DAB concentration, constant among time and organs in the marine bivalves and in cyanobacteria, may suggest that the molecule could be ubiquitous in primary producers or bacteria and/or constitutive of biological matrix, and also require in-depth studies. Similarly, the neurotoxicity of BMAA isomers is almost unknown and that of BMAA is incomplete. The acquisition of new toxicological data is mandatory to characterize the risk and to define toxicological reference values. Finally, the human health risk assessment requires the acquisition of a larger amount of data concerning the presence of total BMAA and isomers in marine and freshwater organisms. This could be done via monitoring programs over several seasons and countries, as well as some case-controlled epidemiological studies in areas of high incidence of ALS combined with nutrition surveys.

## 5. Materials and Methods: Methodology for the Scientific Literature Review

### 5.1. The Strategy for the Extensive Literature Search

The literature review was performed according to the EFSA systematic review guidance [74]. The literature databases Scopus and PubMed were consulted to retrieve the studies reporting the occurrence of BMAA and its isomers DAB, AEG and BAMA in environment and aquatic foods. The search was carried out in English with the entire and abbreviated names of BMAA and isomers connected by the Boolean connector “OR”: “BMAA” OR “β-*N*-methylamino-l-alanine” OR “DAB” OR “2,4-Diaminobutyric Acid” OR “AEG” OR “*N*-(2-aminoethyl)glycine” OR “β-amino-*N*-methyl-alanine” OR “BAMA. The entire names or abbreviations of the toxin and its isomers were combined with the words describing the matrix by the Boolean operator “AND” : AND “Lake” OR “pond” OR “reservoir” OR “sea” OR “ocean” OR “drinking” OR “freshwater” OR “water for aquatic environments and “accumulation” OR “bioaccumulation” OR “food web” OR “shellfish” OR “mussel” OR “oyster” OR “shrimps” OR “clam” OR “seafood” OR “fish for aquatic food sources for humans. The search was performed in “ALL FIELDS”, but we used the Boolean operator “AND NOT” with the terms « DAB » OR « AEG » in the author input field. The search did not include a date of publication constraint but was stopped in November 2016.

### 5.2. The Selection Process of Scientific Papers

The entire selection process is synthetized in Figure 1.

#### 5.2.1. First Screening of Articles

The search resulted in 1228 records for the presence of BMAA and/or isomers in the aquatic environment and 1043 records for the aquatic food sources (Figure 1). A first check for duplicates was done both in EndNote and manually; all duplicates were removed. The first screening of papers was carried out on titles and abstracts according to several inclusion criteria. The study had to: (i) be written in English; (ii) be an original paper and not a review or a book chapter; and (iii) report a concentration of BMAA, DAB, AEG or BAMA in aquatic environments or aquatic species consumed by humans with the data acquired in situ and not in the laboratory (Figure 1). When the abstract of papers did not explicitly describe the work, the full text was read for the first screening step. After this first screening, 17 papers met the inclusion criteria for the occurrence of BMAA and isomers in the environments and 24 in human food (Figure 1).

#### 5.2.2. Second Screening of Articles

The full content of the selected papers was analyzed for a secondary screening with criteria principally based on the analytical methods used for BMAA and isomers detection and quantification. The existence of several BMAA isomers that can be confused with each other during the analysis makes the selection of methods particularly drastic and important. The papers were classified in three categories, A, B and C, depending on the number of qualitative and quantitative analytical criteria fulfilled:

- Qualitative criteria:
  - The indication of the retention time that makes it possible to discriminate BMAA from its isomers; this criterion is strengthened by the monitoring of specific transitions as mentioned below;
  - The indication of the LOD;
  - The LC-MS/MS monitoring of specific transitions for BMAA (*m*/*z* 119 > 88, 76 or 459 > 258 for BMAA without and after derivatization with AQC) and its isomers (*m*/*z* 119 > 101, 74 or 459 > 188 for DAB without and after derivatization with AQC; *m*/*z* 459 > 214 for AEG after derivatization with AQC) to better differentiate them from one another. The ion ratio was an optional and additional criterion allowing the identification of the molecules (e.g., 88/102 and 76/102 for BMAA; 101/102 and 74/102 for DAB; or 258/119, 188/119 and 214/119 for BMAA, DAB and AEG after derivatization by AQC);
- Quantitative criteria:
  - The indication of the LOQ in the matrix (e.g., water and biological matrix);
  - The calculation of the recovery rate during extraction of BMAA or its isomers in the matrix, with the associated standard deviation;
  - Other additional and optional criteria improving the reliability of the analytical method such as the use of radio-labeled internal standard, information concerning the linearity, specificity of the method etc.

When qualitative and quantitative criteria were satisfied, with records providing a description and sufficient information on the analytical method used, a score of A, “reliable without restriction and validated method”, was assigned (4 references for aquatic environments and 8 for food sources, Figure 1). When qualitative criteria were satisfied but not the quantitative ones (not determined in the study or incomplete information given) a score of B, “reliable with restriction (reliable identification but not fully characterized method)”, was assigned (4 references for aquatic environments and 10 for food sources, Figure 1). When neither qualitative nor quantitative criteria were met, a score of C = “not reliable according to the study description”, was assigned (9 references for aquatic environments and 6 for food sources, Figure 1). These criteria induced the consideration of some studies as not reliable despite a rigorous analytical development. The lack of quantitative parameters such as the calculation of the recovery rate during extraction by spiking the matrix with a standard or the absence of LOQ prevented us from considering those studies as fully reliable, for ensuring a fair comparison. For all the above-mentioned reasons LC-FLD, LC-UV, GS-MS, or LC-MS methods were considered as not specific enough to unequivocally distinguish BMAA from its isomers. Even in the case of LC-MS/MS methods, the transitions monitored had to be specific to BMAA and each isomer to distinguish them from each other. When mentioned, the ion ratio reported for the monitored product ions in tandem MS was an additional confirmatory criterion. This level of information is necessary for a reliable analysis ensuring no false positive results and overestimation of the BMAA and isomers concentration in matrix due to potential interfering compounds. It is important to notice that studies using the derivatization of BMAA and its isomers were considered as soon as a LC-MS/MS analysis was associated.

### 5.3. Data Reporting

Most data on BMAA and isomers concentrations in samples of fresh, brackish or marine waters are expressed in µg per unit of dry (μg g^−1^ DW) or fresh (μg g^−1^ FW) weight of phytoplanctonic or phytobenthic materials. When the volume of sampling was not indicated, it remained impossible to express these data per unit of volume, which limits comparison between studies. Free, protein-bound (soluble-bound or precipitated-bound) or total (free + protein-bound) BMAA and isomers in organisms were reported as the minimum, maximum and mean concentrations in edible parts of the species, depending on data available in the studies. Data were reported in mg kg^−1^ FW. When concentrations were expressed by authors in mg kg^−1^ DW, an estimation in mg kg^−1^ FW was performed under the assumption of various percentages of water in tissues depending on taxa: 83.0% for oysters, 83.2% for mussels, 78.4% in crab meat, and 83.0% in crayfish [75]. As a water percentage in fish tissues was not available for every species, we estimated a mean value (i.e., 74.8%) based on available data [75] concerning various species (witches *Glyptocephalus cynoglossus* mean of 82.1%, plaice *Pleuronectes platessa* L. mean of 80.7%, herring *Clupea harengus* mean of 65.3%, and Baltic herring *Clupea harengus membras* mean of 71.2%). In the absence of information on the water content of the shark cartilage, values remained in mg kg^−1^ DW.

## Figures and Tables

**Table 1:** Data related to the contamination of aquatic ecosystems with BMAA and its isomers.

Type of Sample	Type and Number of Samples	Origins	Concentrations of BMAA and Isomers, Reported in µg g^−1^ DW of Phytoplankton Biomass or in µg L^−1^ of Water Sample			LOD	LOQ	Reference	Score
			BMAA	AEG	DAB				
Water	Lake water (*n =* 12)	Canada	Fbmaa ^(1)^ ND-0.3 μg L^−1^	fAEG ^(1)^ ND-0.08 μg L^−1^	fDAB ^(1)^ ND-0.04 μg L^−1^	in µg L^−1^ BMAA 0.008 AEG 0.009 DAB 0.007	in µg L^−1^ BMAA 0.02 AEG 0.03 DAB 0.02	[36]	A
	Finjasjön Lake	Sweden	tBMAA ND-6 ng g^−1^ DW	–	–	BMAA ^(2)^ 0.8 µg L^−1^	BMAA ^(2)^ 0.8 µg L^−1^	[48]	B
	Lake water (*n =* 3)	China	fBMAA ND pbBMAA ND	–	fDAB 0.4–3.8 ng g^−1^ WW pbDAB ND	BMAA 2 pg DAB 5 pg	–	[49]	B
Cyanobacteria	Cyanobacteria	NA	fBMAA ND tBMAA ND	–	–	In µg g^−1^ DW fBMAA 0.2–1 tBMAA 2.5–20	–	[21]	A
	Lagoon periphyton	France	tsBMAA 1.3–4.3 μg g^−1^ DW	tsAEG ND-0.8 μg g^−1^	tsDAB 1.3–4.8 μg g^−1^ DW fDAB: mean 0.49 μg g^−1^ DW	0.23 µg g^−1^ DW	–	[24]	A
	Lagoon seston	France	Micro sbBMAA mean 0.49 μg g^−1^ DW Zoo: sbBMAA mean 0.63 μg g^−1^ DW	Micro ND Zoo ND	Micro sbDAB mean 0.69 μg g^−1^ DW Zoo sbDAB mean 0.92 μg g^−1^ DW	0.23 µg g^−1^ DW	–	[24]	A
	Baltic sea blooms (*n =* 4)	Sweden Finland	fBMAA ND pbBMAA ND	–	–	In µg g^−1^ fBMAA 1 tBMAA 4	–	[25]	A
	Cyanobacterial scums, urban water bodies (*n =* 21)	Netherlands	fBMAA ND-42 μg g^−1^ DW (9/21) pbBMAA ND	–	tDAB ND-4 μg g^−1^ DW (2/21) pbDAB ND	–	–	[34]	B
	Cyanobacteria Baltic Sea, Askö Island	Sweden	tBMAA 2.3–15 ng g^−1^ DW	–	–	70 fmol	–	[40]	B

^(1)^ No acidic extraction (6 N HCl) is mentioned in the material and method section. ^(2)^ Limits of detection/quantification determined for matrices other than water; sb = soluble bound; pb = precipitated bound; f = free; ts = total soluble; t = total; NA = data not available; NQ = not quantified; ND = not detected; DW = dry weight; WW = wet weight; Micro = microalgae-dominated fraction of the seston; Zoo = zooplankton-dominated fraction of the seston.

**Table 2:** Concentrations (mg kg^−1^ FW) ^1^ of free, bound or total BMAA and isomers (DAB, AEG) in edible parts of aquatic organisms reported in A graded studies. When concentrations were reported in mg kg^−1^ DW by the authors, an estimate in mg kg^−1^ FW was calculated. The concentration ranges are indicated for all the samples analyzed.

Type of Sample	Species	Origins	Concentrations (mg kg^−1^ FW) ^(1)^ (Number of Positive Samples/Number of Samples)			LOD	LOQ	Reference
			BMAA	AEG	DAB			
Bivalves	Mussels *Mytilus galloprovincialis*	Thau Lagoon, Mediterranean sea, France	fBMAA ND-0.2 (16/34) tsBMAA max 1.65, mean 0.68 (34/34)	fAEG ND-0.05 (5/34) tsAEG max 0.2 (31/34)	fDAB ND-1.05 (34/34) tsDAB max 1.8, mean 1.22 (34/34)	–	0.15 DW	[24]
	Mussels *Mytilus galloprovincialis and Mytilus edulis*, Oysters *Crassostrea gigas*	Channel, Atlantic, Mediterranean Sea, France	tsBMAA 0.07–1.13 (74/74) tsBMAA 0.03–0.41(23/23)	ND ND	Mussels and oysters: tsDAB 0.2–4.84 (97/97)	–	BMAA: 0.45 DW DAB: 0.15 DW	[42]
	Mussels *Mytilus sp* Oysters *Ostrea edulis*, *Crassostrea gigas*	North Atlantic, Sweden west coast, Greece, France	tBMAA 0.08–0.9 (6/6) tBMAA 0.1–0.66 (4/4)			<0.01 ^(2)^	<0.01 ^(2)^	[50]
	Oysters *Crassostrea virginica*	Louisiana Mississipi	tBMAA 1.5–8 (12/12) tBMAA 1.2–1.7 (3/3)			0.5 ^(3)^	1.7 ^(3)^	[52]
	Mussels *Mytilus galloprovincialis*	Thau Lagoon, Mediterranean Sea, France	fBMAA < 0.34 (4/11) tsBMAA 0.64–2.45 (11/11)	fAEG < 0.08 (3/11) tsAEG 0.1–0.2 (11/11)	fDAB 0.08-1.2 (11/11) tsDAB 0.6–1.6 (11/11)	–	0.15 DW	[23]
	Oysters *Crassostrea gigas*		fBMAA < 0.08 (1/8) tsBMAA 0.5–1.8 (8/8)	fAEG ND (0/8) tsAEG 0.1–0.3 (8/8)	fDAB 0.03–0.6 (8/8) tsDAB 0.6–1.5 (8/8)	–	0.15 DW	[23]
	Mussels	Western coast of Sweden	tBMAA 0.27–1.6 (4/4)				0.15	[53]
	Mussels *Mytilus edulis* *Mytilus edulis platensis* *Perna Canaliculus* Scallops *Placopecten magellanicus*	Scandinavia South America Australia US	fBMAA ND (0/6) tBMAA 0.28–0.59 (6/6) fBMAA ND-0.38 (5/12) tBMAA 1.69–7.08 (12/12) fBMAA ND-0.38 (1/3) tBMAA 0.55–1.14 (3/3) fBMAA 0.18–0.46 (3/3) tBMAA 1.12–1.46 (3/3)			0.10 ^(4)^	0.15 ^(4)^	[54]
Crustaceans	Shrimps *Caridea sp* Crayfish *Astacus leptodactylus*	North Atlantic, Sweden Turkey, Sweden	tBMAA 0.11–0.46 (6/6) tBMAA ND (0/6)			<0.01 ^(2)^	<0.01 ^(2)^	[50]
	Blue crabs *Callinectes sapidus*	Florida	tBMAA 1.08–3.02 (5/5)			0.5 ^(3)^	1.7 ^(3)^	[52]
	Crabs *Cancer pagarus* *Portunus haani* Crayfish *Procambrus claarki* Shrimps *Pandalus borealis*	Ireland, North Atlantic Vietnam China Greenland, North Atlantic	tBMAA detected, NQ (1/1) tBMAA ND (0/1) tBMAA ND (0/1) tBMAA ND (0/3)			0.10 ^(3)^	0.15 ^(3)^	[54]
Fish	Plaice *Pleuronectes platessa*, Herring *Clupea harengus* Char *Salvelinus alpinus* Salmon *Salmo salar* Cod *Gadus morhua* Perch *Perca fluviatilis*	North Atlantic Baltic Sea Baltic Sea Sweden Norway Norway North Atlantic Sweden	tBMAA 0.01–0.02 (3/3) tBMAA ND-0.02 (1/3) tBMAA ND-0.01 (1/3) tBMAA ND (0/4) tBMAA ND (0/4) tBMAA ND (0/4)			<0.01 ^(2)^	<0.01 ^(2)^	[50]
	Atlantic salmon *Salmo salar* Sea bass *Dicentrarchus labrax* Sea bream *Sparus aurata* Whitefish *Coregonus sp*, Pike perch *Sander lucioperca*, Sea trout *Salmo truttae*	Norway Italia Greece Sweden Sweden Baltic Sea Sweden Bothnain Sea	tBMAA ND (0/1) tBMAA ND (0/1) tBMAA ND (0/1) tBMAA ND (0/1) tBMAA ND (0/1) tBMAA ND (0/2)			0.10 ^(4)^	0.15 ^(4)^	[54]
	Shark cartilage powder, variety of shark species not identified	Commercial food supplements, from 7 manufacturers	**(In mg kg^−1^ DW)** tBMAA ^(5)^ 74.8–352.2 (15/16)	**(In mg kg^−1^ DW)** tAEG ^(5)^ 1298.4–1765.1 (16/16)	**(In mg kg^−1^ DW)** etDAB ^(5)^ 69.2–1483.4 (16/16)	(in pg L^−1^) BMAA 1.1 AEG 1.2 DAB 0.8		[55]

^(1)^ Except for Mondo et al. [55] whose results are reported in mg kg^−1^ DW. ^(2)^ LODs and LOQs determined for a crayfish matrix. ^(3)^ LODs and LOQs determined for a sea hare matrix. ^(4)^ LODs and LOQs determined for a mussel matrix. ^(5)^ Two methods were used in this study but only samples analyzed by UPLC-MS/MS are considered here. Sb = soluble bound; pb = precipitated bound; f = free; ts = total soluble; t = total; NA = data not available; NQ = not quantified; ND = not detected; DW = dry weight; FW = wet weight.

**Table 3:** Concentrations (mg kg^−1^ FW) ^(1)^ of free, bound or total BMAA and isomers (DAB, AEG) in edible parts of aquatic organisms reported in B graded studies. When concentrations were reported in mg kg^−1^ DW by authors, an estimate in mg kg^−1^ FW was calculated.

Type of Sample	Species, Number of Sample	Origins	Concentrations (mg kg^−1^ FW) ^(1)^			LOD	LOQ	Reference
			BMAA	AEG	DAB			
Bivalves	Mussels *Mytilus edulis* Oyster *Ostrea edulis*	Baltic Sea	tBMAA 0.02–0.03 (3/3) tBMAA ND-0.02 (3/3)	–	–	70 fmol	–	[40]
	Cockles *Cerastoderma edule* samples of 30 individuals	Ria de Aveiro Ria Formosa Portugal	pbBMAA 0.018–0.081 (9/9) pbBMAA ND-0.1 (5/10)	–	–	0.05 ng	0.05 ng	[56]
	Mussels *Mytilus edulis*	Canada	tBMAA 0.19–0.24 (9/9)	NQ	NQ	20 ng g^−1^ DW	–	[57]
	Mollusks (29 species) 68 samples Mussel *Mytilus coruscus* Razor clam *Solen strictus* Gastropod *Neverita didyma* Other marine species: *Mytilus galloprovincialis*, *Crassostrea* sp., *Perna viridis*, *Antigona lamellaris*, *Atrina pectinata*, *Meretrix lusoria*, *Periglypta petechialis*, *Chlamys farreri*, *Mactra chinensis*, *Ruditapes philippinarum*, *Sinonovacula constrita*, *Tegillarca granosa*, *Haliotis discus hannai*, *Turritella bacillum*, *Natica maculosa*, *Batillaria zonalis*, *Moerella iridescens*, *Scapharca subcrenata*, *Mactra chinensis*, *Volutharpa ampullacea*, *Neptunea cumingii*, *Arca inflata*, *Merceneria merceneria*, *Rapana venosa*, *Argopecten irradians*, *Mimachlamys nobilis*, *Gafrarium tumidum*	Sampling in aquaculture zones and markets from 10 cities along the Chinese coast, and in situ sampling of gastropods	fBMAA (5/68) in: fBMAA 0.45 (1/2) fBMAA 0.66 (1/1) fBMAA 0.99–3.97 (3/5) For all samples pbBMAA ND (0/68)	For all samples fAEG and pbAEG ND	For all samples pbDAB ND fDAB (53/68), in 23 marine species 0.05–2.65	BMAA: 0.31 AEG: 0.10 DAB: 0.013	–	[58]
Crustaceans	Lobster *Panulirus sp* muscle tail	Florida	Boiled flesh: tBMAA ^(2)^ 0.70–6.94 (4/4) Fresh flesh: fBMAA ND-0.08 (1/2) pbBMAA 0.42–2.20 (1/2)	–	Boiled flesh: tDAB ^(2)^ 1.10–8.33 (4/4) Fresh flesh: fDAB 0.17–0.21 (2/2) pbDAB 0.01–0.08 (2/2)	BMAA: 48 fmol DAB: 26 fmol	BMAA: 0.48 fmol DAB: 0.26 fmol	[43]
	Blue crab *Callinectes sapidus* meat of claws	East Atlantic	tsBMAA ND-24.8 (2/3) pbBMAA ND-10.80 (1/3)		tDAB 11.57–15.67 (3/3) pbDAB ND-2.91 (1/3)	–	–	[44]
Fish	Smelt *Osmerus eperlanus* Turbot *Scophthalmus maximus* Herring *Clupea harengus* Common whitefish *Coregonus laveratus* Pike-perch *Sander lucioperca*, fourhorn sculpin *Triglopsis quadricornis*, salmon *Salmo salar*	Baltic Sea	tBMAA in muscles 0.003–0.05 (3/3) 0.001–0.003 (2/3) 0.002 (1/3) 0.007–0.014 (2/3) ND (0/3 for all species)	–	–	70 fmol	–	[40]
	Bream *Abramis brama* Perch *Perca fluviatilis* Pike *Esox Lucius* Pike-perch *Sander lucioperca* Roach *Rutilus rutilus* Ruffe *Gymnocephalus cernua* Tench *Tinca tinca*	Sweden, freshwater lake	tBMAA in muscles 0.00002 ± 0.00006 (9/32) 0.00002 (1/29) 0.00001 (1/22) 0.0003–0.00013 (3/29) 0.00004 (1/24) 0.0008 ± 0.0008 (4/15) 0.0014 (1/15)	–	–	0.8 ng mL^−1^	–	[48]
	Carp *Cyprinus carpio*	New Hampshire	f+pbBMAA 0.25 (1/1) in muscles	–	f+pbDAB ND (0/1)	(in fmol) BMAA 48 DAB 26	(in fmol) BMAA 0.48 DAB 0.26	[51]
	Fin of 1 species, Tiger shark *Galeocerdo cuvier* evaluated by LC-MS/MS	Atlantic and Pacific	tBMAA 19.2–33.15 (4/4)	–	–	HPLC-FD: 2.7 ng	HPLC: 7.0 ng	[59]
	A single fin sample of the hammerhead shark *Sphyrna mokarran* evaluated by LC-MS/MS	Biscayne Bay Florida Bay	tBMAA identified, NQ	–	–	HPLC-FD: 2.7 ng	HPLC: 7.0 ng	[60]

^(1)^ Two methods were used in this study but only samples analyzed by UPLC-MS/MS are considered here. ^(2)^ Sum of values for hydrolysed supernatant and pellet. Sb = soluble bound; pb = precipitated bound; f = free; ts = total soluble; t = total; NA = data not available; NQ = not quantified; ND = not detected; DW = dry weight; FW= wet weight.
