# Correction: Occurrence of β-*N*-methylamino-l-alanine (BMAA) and Isomers in Aquatic Environments and Aquatic Food Sources for Humans. *Toxins* 2018, *10*, 83

**DOI:** 10.3390/toxins10050191

**Published:** 2018-05-10

**Authors:** Emilie Lance, Nathalie Arnich, Thomas Maignien, Ronel Biré

**Affiliations:** 1 UMR SEBIO, Bat 18, Campus du Moulin de la Housse, BP 1039, 51687 REIMS CEDEX 2, France; 2 ANSES—French Agency for Food, Environmental and Occupational Health & Safety, Direction de l’Evaluation des Risques, 14 rue; Pierre et Marie Curie, 94701 Maisons-Alfort, France; nathalie.arnich@anses.fr (N.A.), thomas.maignien@anses.fr (T.M.); 3 Université Paris-Est, ANSES, Laboratory for Food Safety, F94701 Maisons-Alfort, France; ronel.bire@anses.fr

The authors wish to correct the citation of some references in this paper [1].

In the second paragraph of the Introduction, “except for the nitrogen concentration in the medium that might modify BMAA biosynthesis [29,30] should be replaced with “except for the nitrogen concentration in the medium that might modify BMAA biosynthesis [29]”. Also, “but not for all species [17]” should be replaced with “but not for all species [30]”. Further, “its isomers DAB and AEG [15–17]” should be replaced with “its isomers DAB and AEG [24,30]”.

In the Conclusion, “some references we graded B have not been taken into account [21,40,52,54,57,58,60,62]” should be replaced with “some references we graded B have not been taken into account [28,43,44,48,51,58–60]”. Also, “the reported data on BMAA content in fish mainly derived from studies we graded C [25,32,34]” should be replaced with “the reported data on BMAA content in fish mainly derived from studies we graded C [32,39]”.

The changes do not affect the scientific results. The manuscript will be updated and the original will remain online on the article webpage. We apologize for any inconvenience caused to our readers.
